# Genomics insights into a *Mycobacterium pinnipedii* isolate causing tuberculosis in a captive South American sea lion (*Otaria flavescens*) from Italy

**DOI:** 10.3389/fmicb.2023.1303682

**Published:** 2023-12-21

**Authors:** Patricia Alba, Andrea Caprioli, Cristiano Cocumelli, Claudia Eleni, Elena Lavinia Diaconu, Valentina Donati, Angela Ianzano, Luigi Sorbara, Fiorentino Stravino, Natalino Cerini, Maria Beatrice Boniotti, Mariagrazia Zanoni, Alessia Franco, Antonio Battisti

**Affiliations:** ^1^Department of General Diagnostics, Istituto Zooprofilattico Sperimentale del Lazio e della Toscana “M. Aleandri”, Rome, Italy; ^2^Azienda Sanitaria Locale Roma 6, Servizi Veterinari, Rome, Italy; ^3^Istituto Zooprofilattico Sperimentale della Lombardia e dell’Emilia-Romagna, Brescia, Italy

**Keywords:** *Mycobacterium pinnipedii*, tuberculosis, marine mammals, zoo, zoonosis, genomics, virulomevirulome, resistome

## Abstract

Tuberculosis (TB) affects humans and other animals, and it is caused by bacteria within the *Mycobacterium tuberculosis* complex (MTBC). In this study, we report the characterisation of *Mycobacterium pinnipedii* that caused a TB case in a sea lion (*Otaria flavescens*) kept in an Italian zoo. The animal died due to severe, progressive disorders involving the respiratory and gastro-enteric systems and the skin. At necropsy, typical gross lesions referable to a TB generalised form were found. In particular, nodular granulomatous lesions were detected in the lungs and several lymph nodes, and colonies referable to *Mycobacterium* spp. were isolated from lung, mesenteric, and mediastinal lymph nodes. The isolate was identified by PCR as a MTBC, had a spoligotype SB 1480 (“seal lineage”), and was characterised and characterised by whole-genome sequencing analysis confirming that the MTBC involved was *M. pinnipedii*. The analysis of the resistome and virulome indicated the presence of macrolide and aminoglycoside resistance genes intrinsic in *M. tuberculosis* [*erm-37* and *aac*(2′)-Ic] and confirmed the presence of the region of difference 1 (RD1), harbouring the *esx*A and *esx*B virulence genes, differently from its closest taxon, *M. microti*. As for other MTCB members, *M. pinnipedii* infection can spill over into non-pinniped mammalian species; therefore, zoological gardens, veterinary practitioners, and public health officers should be aware of the hazard posed by tuberculosis from marine mammals. Since the isolate under study, as well as all available genomes of *M. pinnipedii* investigated in this study retains almost all the *M. tuberculosis* virulence genes, it could indeed cause infection, lesions, and disease in other animal species, including humans.

## 1 Introduction

Tuberculosis (TB) affects humans and other animals, and it is caused by bacteria within the *Mycobacterium tuberculosis* complex (MTBC). MTBC members belong to the family Mycobacteriaceae and are Gram-positive, acid-fast bacilli. The taxonomy of organisms in the MTBC is in constant evolution [World Organisation for Animal Health (WOAH), 2022]. Based on the current knowledge, the MTBC is comprised of nine human-adapted lineages (L), representing *M. tuberculosis sensu stricto* (L1–4 and L7–8) and *M. africanum* (L5–6 and L9), plus different animal-associated lineages including *M. bovis*, *M. caprae*, *M. microti*, and *M. pinnipedii* (Riojas et al., 2018; Silva et al., 2022; Vågene et al., 2022).

All MTBC members have almost identical genome sequences/genotypes, with more than 99.95% nucleotide identity, and although all mammalian species are considered susceptible to tuberculosis, their host tropism and ability to cause disease differ significantly (Silva-Pereira et al., 2019). Single-nucleotide polymorphisms (SNPs) and deletions of genomic regions ranging from 2 to 12.7 Kb, denominated ‘regions of difference (RDs)’, allow for species differentiation [Cousins et al., 2003; Silva-Pereira et al., 2019; World Organisation for Animal Health (WOAH), 2022].

*Mycobacterium pinnipedii* was first described in pinnipeds in 1913 (Blair, 1913), but it was not until 2003 that the causal agent was recognised as a novel member of the MTBC, now known as *M. pinnipedii* (Cousins et al., 2003). *Mycobacterium pinnipedii* has been mostly isolated from pinnipeds (seals and sea lions) worldwide, more often in captive animals (Cousins et al., 2003; Kiers et al., 2008; Moser et al., 2008; Kriz et al., 2011; Boardman et al., 2014; de Amorim et al., 2014; Martins Melo et al., 2019; Roe et al., 2019; Silva-Pereira et al., 2019; Macedo et al., 2020; Lindsay and Gray, 2021). However, the host range for *M. pinnipedii* infection appears to be rather broad, with infections reported in multiple non-marine mammal species (Moser et al., 2008; Jurczynski et al., 2011; Loeffler et al., 2014; Roe et al., 2019), including humans (Cousins et al., 2003; Kiers et al., 2008; Macedo et al., 2020) and non-human primates, often resulting in clinical tuberculosis (Cousins et al., 2003; Cousins, 2006). The virulence of the organism has also been confirmed by experimental infections (Cousins et al., 2003; Cousins, 2006) and genomic studies on ancient bacteria recovered from archaeological human skeletal remains from South America (Bos et al., 2014; Vågene et al., 2022).

Direct contact with pinnipeds infected with *M. pinnipedii*, in particular those kept in zoos and marine parks, is the main source of infection for other animals and humans (Silva-Pereira et al., 2019) although waters and shores possibly contaminated by infected pinnipeds have also been associated with transmission events of *M. pinnipedii* to grazing cattle (Loeffler et al., 2014).

Genome sequencing of *M. pinnipedii* isolates and comparative genomic analyses in order to better explore the genetics of these bacteria, including phylogenesis, virulence, and antimicrobial resistance traits, has been occasionally performed, contributing to a better understanding of *M. pinnipedii* epidemiology, zoonotic potential, and MCTB past history (Bos et al., 2014; Riojas et al., 2018; Silva-Pereira et al., 2019; Macedo et al., 2020; Vågene et al., 2022).

The objectives of the present study were to: describe the TB case in a captive sea lion and its aetiology; perform in-depth genomics characterisation of the isolate by whole-genome sequencing (WGS) and bioinformatics in order to gain insight into its zoonotic potential through the assessment of its main genomic traits (e.g., those associated with virulence and antimicrobial resistance); and phylogenetically compare it with other *M. pinnipedii* genomes available in public repositories.

## 2 Materials and methods

### 2.1 Sea lion origin and clinical picture

In 2021, a dead 18-year-old male South American sea lion (*Otaria flavescens*) was sent to the Istituto Zooprofilattico Sperimentale del Lazio e della Toscana “M. Aleandri” (IZSLT) to ascertain the causes of death. The animal had been kept at a zoo located in Central Italy. The zoo covers an area of 40 ha and contains approximately 350 animals of 36 different species (mammals, birds, and reptiles) at the time of the case, including a population of 14 pinnipeds (all born in captivity) kept in an open-air basin. The pinniped species included four *Otaria flavescens*, four *Zalophus californianus*, three *Phoca vitulina*, and three *Halichoerus grypus*. The sea lion was born in a Belgian zoo in 2003, was temporarily relocated to a Portuguese zoo, and arrived at the Italian zoo in 2005. To the best of our knowledge, it had never been tested for TB (direct or indirect) before its death.

The animal died following several days of progressive signs characterised by diarrhoea, inappetence, sensory depression, severe subcutaneous emphysema, and difficulty in breathing. No other pinnipeds were reported to be sick at the time of the death, and subsequently, no other pinnipeds died at the zoo.

### 2.2 Pathology, sample collection, histopathology, and bacteriology

A complete examination and necropsy of the sea lion’s corpse were performed. During necropsy, samples of several biological specimens were collected; parts were fixed in 10% neutral buffered formalin for histopathological examination, and parts were subjected to standard bacteriological analyses. Briefly, for bacteria isolation and identification, tissue/organs were cultured on Columbia agar supplemented with 5% sheep blood (VWR, Belgium) and brain heart infusion broth; following incubation for 24 to 48 h at 37°C under aerobic and microaerobic (10% CO2) conditions, growth colonies were subcultured, and pure colonies were screened using standard techniques including colony morphology, Gram staining, catalase test, oxidase test, and biochemically identified at the species level with API test kits (bioMérieux, France). Intestine content was screened for the presence of *Salmonella* spp. following international recommendations [World Organisation for Animal Health (WOAH), 2022], as previously described (Alba et al., 2013). The lung and several lymph nodes from various apparatus showing suspected TB lesions were also subjected to Ziehl–Neelsen acid-fast staining and cultured using specific solid commercial media for the isolation of *Mycobacterium* spp. (Stonebrink and Lowenstein–Jensen media, Microbiol S.n.c., Italy), following the specific chapter of the WOAH Manual of Diagnostic Tests and Vaccines for Terrestrial Animals 2022 [World Organisation for Animal Health (WOAH), 2022]. Briefly, specimens for culture were processed by removing extraneous material (e.g., fat) using sterile scissors, then tissues were cut into small pieces and homogenised using a stomacher with sterile PBS, followed by a decontamination step using a 2% sodium hydroxide solution for 20 min. The suspension was centrifuged, the supernatant was discarded, and the sediment was used for solid media inoculation.

### 2.3 Mycobacterium molecular identification and genetic characterisation

DNA from suspect colonies referable to *Mycobacterium* spp. was extracted using the QIAamp DNA Mini Kit (Qiagen, Hilden, Germany) following the manufacturer’s protocol and as previously described (Iurescia et al., 2021). Extracted DNA was subjected to multiplex end-point PCR for identification at the genus level and to assess their belonging to MTBC (Kulski et al., 1995). Identification of the MTBC involved was assessed by the amplification of the genomic region of difference RD2, in particular using the RD2^seal^ PCR protocol described by Warren et al. (2006). Spoligotyping, following the protocol described by Kamerbeek et al. (1997), was performed by hybridization onto a spoligotyping membrane (Mapmygenome, Hyderabad, India), where the spacer sequences contained in the direct repeat locus were spotted. Spoligotypes were named in accordance with an international convention by using the www.mbovis.org database (Smith and Upton, 2012).

The MTBC isolates retrieved were also investigated by whole-genome sequencing (WGS) analysis. Libraries for short read pair-end sequencing were prepared using the Nextera XT DNA library preparation kit (Illumina, Inc., San Diego, CA, United States) following the Nextera XT R Guide 150319425031942 and sequenced on an Illumina platform (MiSeq). Quality trimming of the reads was performed using Trimmomatic v0.39 with the following parameters: LEADING:30, TRAILING:30, SLIDINGWINDOW:10:20, and MINLEN:50 (Bolger et al., 2014). Assembly was performed using SPAdes v3.13.0 (Bankevich et al., 2012). The quality of the assembly was addressed using QUAST v5.0.2 (Gurevich et al., 2013). Trimmed raw reads and assemblies were mapped against the *M. tuberculosis* reference sequence H37Rv (NC_000962) by using minimap2 v2.24-r1122 (Li, 2018), samtools v1.12 (Danecek et al., 2021) for sorting and indexing, and IGV v2.5.3 (Thorvaldsdóttir et al., 2013) for visualisation. The interpretation was performed manually by checking the deletions described previously by Brodin et al. (2002) for *M. microti*.

Multilocus sequence typing (MLST) was carried out by using the scheme published in the pubMLST.org database (https://pubmlst.org/organisms/mycobacteria-spp; Jolley et al., 2018) and uploading the complete assembly. Resistance and virulence genes were screened using abricate^1^ with the databases ResFinder (Zankari et al., 2012; updated on 9 November 2022) and vfdb (Chen et al., 2005, updated on 22 April 2022), respectively. Point mutations were investigated using the PointFinder online tool (https://cge.food.dtu.dk/services/ResFinder/ accessed 15 November 2023; Zankari et al., 2017). All the analyses were performed with the following cutoffs: minimum 80% coverage and 80% identity.

The following publicly available raw reads were downloaded for phylogenetic comparison purposes: ERR150046 (chimpanzee bacillus), SRR3745458 (dassie bacillus), ERR234255 and SRR998578 (*M. africanum*), SRR6705904 (*M. bovis*), DRR120409 (*M. caprae*), ERR027298 (*M. microti*), SRR3500411 (*M. mungi*), SRR5642712 (*M. orygis*), ERR970409 (*M. suricattae*), ERR552768, SRR1238557, SRR1238558, SRR1239336, SRR1239337, SRR1239338, SRR1239339, ERR4143898, ERR4143897, SRR7693584, and SRR7693090 (*M. pinnipedii*). For virulence determinant comparison, the following raw reads of *M. microti* were downloaded for further selected bioinformatics analysis: ERR2659163, ERR2659164, ERR2659165, ERR2659167, ERR2659168, ERR2659169, ERR2659170, ERR2659171, and ERR551111.

SNPs were obtained by using Snippy 4.6^2^ with the default parameters (minimum quality of the nucleotide set as 13; minimum coverage set as 10; minimum proportion of those reads that must differ from the reference set as 0.9). The obtained core alignment was used as input for tree generation. The phylogenetic tree was built using RAxML 8.2.12 (Stamatakis, 2014) and the maximum likelihood (ML) algorithm with the ‘GTRCAT’ model and 1,000 bootstrap inferences in order to remain adherent to the tools used in the phylogeny was proposed by Silva-Pereira et al. (2019). The best tree was chosen and re-rooted with the reference strain. The visualisation of the figure was created using iTol (Letunic and Bork, 2021).

## 3 Results

### 3.1 Pathology, histopathology, and bacterial cultures

At necropsy, the animal had severe generalised subcutaneous emphysema and necrotizing and suppurative generalised lymphadenitis with multifocal areas of partial calcification. In the thoracic cavity, in addition to lymphadenitis, there was a severe pleural effusion of serohaemorrhagic fluid, a severe, bilateral, coalescing granulomatous pneumonia, and evidence of suppurative and haemorrhagic tracheitis and bronchitis, with ulcers and fibrino-necrotic plaques on the mucosa. Severe granulomatous lymphadenitis was evident also in the abdominal cavity, with catarrhal and ulcerative enteritis of the first tract of the duodenum and the presence of disseminated miliary nodules in the liver parenchyma.

The main lesions observed at histopathology were characterised by severe granulomatous inflammation, with a high number of foamy macrophages surrounding central areas of necrosis and different degrees of mineral deposits, particularly concentrated in the lung and lymph nodes; similar foci of necrosis were evident in the liver parenchyma but without macrophage infiltration. Ziehl–Neelsen staining performed on lymph nodes of different areas (thorax and abdomen) and lung sections highlighted the presence of a few acid-fast bacteria, morphologically resembling *Mycobacterium* spp.

After approximately 10 weeks of post-inoculation, suspect colonies referable to *Mycobacterium* spp. were detected on TB-specific media from lung, mesenteric, and mediastinal lymph nodes.

Standard cultures for other bacteria were performed on samples (lung, pleural fluid, intestine, mesenteric, and mediastinal lymph nodes) and were positive for the presence of *Pseudomonas aeruginosa* (lung, intestine, mesenteric, and mediastinal lymph nodes) and *Staphylococcus pseudintermedius* (mediastinal lymph nodes), respectively.

### 3.2 Mycobacterium molecular identification and genomics

*Mycobacterium* spp. suspected colonies from both lung and mesenteric and mediastinal lymph nodes were positive at the genus level and for MTBC by multiplex end-point PCR amplification of the Region of Difference RD2 failed, indicating *M. pinnipedii* as the aetiological agent. Spoligotyping analysis revealed the presence of an SB1480 profile, a typical *M. pinnipedii* spoligotype.

The WGS assembly presented a 58X median depth and covers 97.3678% of the *M. tuberculosis* H37Rv genome. The overall quality of the assembly was good, and, in the end, 128 contigs (>500 bp) were obtained.

**Figure 1:**
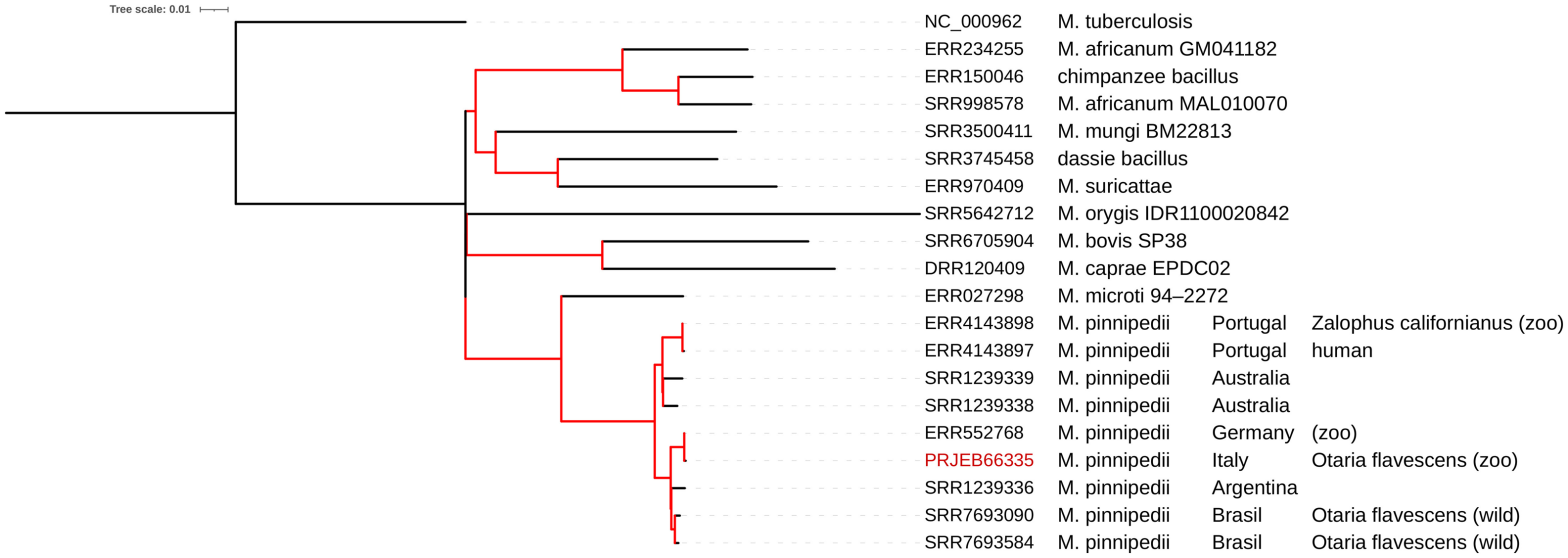
Phylogenetic tree calculated with the ML algorithm for several genomes of the *Mycobacterium* TB complex. The red branches indicate a 100% bootstrap. The tree scale indicates that 0.1 nucleotide substitutions per site correspond to that length in a branch.

The mapping analysis against *M. tuberculosis* H37Rv confirmed the identity as *M. pinnipedii* by revealing the absence (deletion) of the region of difference 2 (RD2 ‘seal’). Moreover, the SNP-based phylogenomics approach (Silva-Pereira et al., 2019) placed our isolate (PRJEB66335) in Cluster 1 of *M. pinnipedii* (Figure 1). The pairwise SNP distances of the isolates located in this cluster ranged from 2 to 84 SNPs of difference, and the genetically closest isolate was *M. pinnipedii* isolated in Germany (ERR552768), with only 2 SNPs of difference, for which the animal species of origin and year of isolation are not reported (Malm et al., 2017; Brites et al., 2018). Isolates other than *M. pinnipedii* showed a pairwise SNP distance greater than 300 SNPs of difference. The SNP calling variant process resulted in the non-inclusion of the following genomes available from international repositories: SRR1238557, SRR1238558, and SRR1239337.

MLST from WGS data indicated that our isolate belongs to sequence type (ST)215, a common ST amongst isolates of the MTBC taxa (Jolley et al., 2018). Only two natural (intrinsic) resistance genes were found using the ResFinder database: *erm-37* and *aac*(2′)-Ic, encoding for macrolide and aminoglycoside resistances, respectively. Chromosomal mutations known to be related to antibiotic resistance were not found. Several mutations have been found in the chromosomal genes as *gyr*A, *gyr*B, or *rpo*B, possibly related to antimicrobial resistance but with unknown effects on the phenotype (Supplementary File S1). Amongst others, an interesting feature is an insertion of 6 bp in the *rpo*B gene, a gene known to be associated with rifampicin resistance in certain versions. It causes a two amino-acid insertion (R849insRED) in an external loop of the protein. Regarding the virulence genes, 139 genes of the MTBC were detected, amongst the 142 genes of *M. tuberculosis* H37Rv (NC_000962), including *esx*A (6 kDa early secretory antigenic target), *esx*B (10 kDa culture filtrate antigen), and the *mbt*A, *mbt*B, *mbt*C, *mbt*D, *mbt*E, and *mbt*F genes, involved in the biosynthesis of the siderophore mycobactin. The virulome of our *M. pinnipedii* strain was very similar to the virulome of the other *M. pinnipedii* genomes we had retrieved from public repositories and analysed in this study. Conversely, all the *M. microti* genomes here analysed lacked *esx*A, *esx*B, and some genes involved in the ESX-1 type VII secretion system as *ecc*A1, *ecc*B1, or *ecc*Ca1 (Supplementary File S1).

The raw reads obtained were submitted to the European Nucleotide Archive (ENA)^3^ under the study accession number PRJEB66335.

## 4 Discussion

In this study, we have reported, for the first time in Italy, a case of tuberculosis from *M. pinnipedii* infection in a South American sea lion (*Otaria flavescens*) who died at an Italian zoo. The isolation of other bacteria (*Pseudomonas aeruginosa* and *Staphylococcus pseudintermedius*) is probably attributable to secondary infections that may have contributed to the clinical picture and to animal death.

The genomic investigation confirmed that the isolate belonged to the taxon *pinnipedii* because of the lack of the RD2 (‘seal’ region). The identification of the taxon as *pinnipedii* was further confirmed by the high similarity with other *M. pinnipedii* genomes in the SNP-based phylogenomics approach, conducted as in the study by Silva-Pereira et al. (2019), which placed the Italian isolate PRJEB66335 in Cluster 1 of *M. pinnipedii* amongst the isolates of South American origin. Indeed, modern *M. pinnipedii* strains appear to be divided into two groups according to geographic locations and possibly host species: modern Cluster 1, comprising isolates of South American origin, and modern Cluster 2, comprising isolates from Australia (Silva-Pereira et al., 2019).

Since the resistome and virulome found in our isolate are considered intrinsic features of *M. tuberculosis*, their presence in *M. pinnipedii* could also be considered intrinsic. No other acquired resistance genes or mutations conferring antimicrobial resistance features were detected by the consensus databases ResFinder/PlasmidFinder. Regarding the insertion with no reported effects in the *rpo*B gene (Zankari et al., 2017; Li et al., 2021; WHO, 2021), since it is located outside the RIF resistance-determining region (RRDR) and outside the pocket where rifampicin fits (Pang et al., 2013; Li et al., 2021), there is no evidence that it can provide a genetic basis associated with a rifampicin resistance phenotype (i.e., silent insertion). However, further consensus evidence is necessary to confirm this point.

Differently from *M. microti*, phylogenetically its closest taxon amongst MTBC (Brites et al., 2018; Silva-Pereira et al., 2019), the *M. pinnipedii* under study also preserves the RD1 region, like all other available *M. pinnipedii* genomes here analysed (Figure 1). RD1 harbours the *esx*A and *esx*b virulence genes, well-known virulence markers of *M. tuberculosis*, that encode ESAT-6 and CFP-10, respectively (Meher et al., 2006). Indeed, the RD1 deletion in *M. microti* has been described as one of the causes of its minor pathogenicity (Brodin et al., 2002), which is also the most relevant mutation associated with attenuation in the *M. bovis* BCG vaccine (Pym et al., 2002). The fact that *M. pinnipedii* conserves the RD region and its virulence genes should be considered a further marker and indicator of its zoonotic potential.

The infection caused by *M. pinnipedii* has been described in pinnipeds worldwide, particularly in captive animals, but since 2018, to our knowledge, new cases have no longer been reported in captive or wild pinnipeds from Europe (Macedo et al., 2020). The 18-year-old sea lion did not show any signs compatible with TB, such as respiratory signs or enlargement of superficial lymph nodes, before 2021. The source of the infection remains unknown, but since no recent new introductions of pinnipeds were reported at the zoo (the last one was in 2017), it is possible that the infection was chronic and long-lasting. The gross and histological lesions found are similar to those described for other pinniped species (Cousins et al., 2003; Roe et al., 2019; Lindsay and Gray, 2021). In particular, lesions detected were characterised by lung and lymph nodes, granulomatous inflammation and pleural effusion, tracheitis, and bronchitis, further supporting respiratory secretions as the probable main pathway of transmission in pinnipeds (Cousins et al., 2003). Interestingly, the genomics data indicated that amongst the available genomes, our *M. pinnipedii* isolate was most similar to *M. pinnipedii* (host species of isolation and year of isolation unknown) from Germany (Malm et al., 2017; Brites et al., 2018); hence, a possible common origin of the infection chain might be speculated. The possibility that the animal had long-lasting sub-clinical signs is concerning, since it suggests that this zoonotic pathogen might circulate undetected in captive pinnipeds in the zoo network for a long period of time. More in general, this situation may be favoured amongst captive pinnipeds by the lack of validated intra-vitam tuberculosis diagnostic methods available in marine mammals.

Considering the risks, the local competent authority (CA) soon implemented risk mitigation action for the personnel and visitors and a surveillance programme amongst the pinnipeds and other mammals at the zoo, which is still ongoing, based on clinical assessment and direct testing (specific PCR for MTBC and TB cultures) on faeces and saliva samples. However, at the time of the article submission, the CA has not reported any new cases of animal TB infection or disease, nor has it notified any human cases of tuberculosis amongst the animal husbandry staff at the zoo.

## 5 Conclusion

Although MTBC comprises host-adapted taxa, inter-species transmission between wild, captive, and domesticated animals and humans has been frequently reported, posing a worldwide threat to both human and animal health (Vågene et al., 2022).

People who can come into close contact with animals infected by *M. pinnipedii* (mainly zookeepers), either through direct contact or exposure to a contaminated environment, should be aware of the hazards posed by tuberculosis. The bacterial pathogen detected in the case under study (similarly to all other *M. pinnipedii* genomes here investigated) has virulence traits associated with zoonotic potential reportedly claimed for past descriptions of *M. pinnipedii* infection and disease cases and harbours almost the same key virulence genes as *M. tuberculosis*. Our findings highlight the need for awareness and constant vigilance of the zoonotic hazard posed to any person in close contact with captive pinnipeds, even in the absence of suspected TB signs (e.g., respiratory signs).

## Data availability statement

The datasets presented in this study can be found in online repositories. The names of the repository/repositories and accession number(s) can be found in the article/Supplementary material.

## Ethics statement

Ethical approval was not required for the studies involving animals in accordance with the local legislation and institutional requirements because it was not applicable since we reported a case of tuberculosis on a dead animal received for necropsy. Written informed consent was obtained from the owners for the participation of their animals in this study.

## Author contributions

PA: Writing – original draft, Conceptualization, Data curation, Formal analysis, Investigation, Methodology, Software, Supervision, Validation, Visualization, Writing – review & editing. AC: Conceptualization, Methodology, Supervision, Validation, Visualization, Writing – original draft, Writing – review & editing, Formal analysis. CC: Conceptualization, Investigation, Methodology, Supervision, Visualization, Writing – review & editing. CE: Investigation, Methodology, Supervision, Visualization, Writing – review & editing. ED: Data curation, Investigation, Methodology, Software, Visualization, Writing – review & editing. VD: Data curation, Investigation, Methodology, Visualization, Writing – review & editing. AI: Investigation, Methodology, Visualization, Writing – review & editing. LS: Investigation, Visualization, Writing – review & editing. FS: Investigation, Visualization, Writing – review & editing. NC: Writing – review & editing, Conceptualization, Visualization. MB: Data curation, Investigation, Methodology, Software, Visualization, Writing – review & editing. MZ: Investigation, Methodology, Visualization, Writing – review & editing, Data curation. AF: Conceptualization, Data curation, Methodology, Resources, Supervision, Validation, Visualization, Writing – review & editing. AB: Conceptualization, Data curation, Methodology, Resources, Supervision, Validation, Visualization, Writing – review & editing.

## Footnotes

^1^ https://github.com/tseemann/abricate

^2^ https://github.com/tseemann/snippy

^3^ http://www.ebi.ac.uk/ena
